# Brazilian Survey on Preventive Actions for the Population With Access to Primary Healthcare: Inefficient Spending in a Country in Economic Crisis

**DOI:** 10.34172/ijhpm.2021.94

**Published:** 2021-08-30

**Authors:** Patrícia Sampaio Chueiri, Marcelo Rodrigues Gonçalves, Lisiane Hauser, Sotero Mengue, Milena Agostinho, Rudi Roman, Lucas Wollmann, Anna Dilda, Rodrigo Aquino Martins da Silva, Erno Harzheim

**Affiliations:** ^1^School of Medicine, Faculdade Israelita de Ciências da Saúde Albert Einstein, São Paulo, Brazil.; ^2^Graduate Program in Epidemiology, TelehealthRS Project, School of Medicine, Universidade Federal do Rio Grande do Sul (UFRGS), Porto Alegre, Brazil.; ^3^Universidade de Caxias do Sul (UCS), Caxias do Sul, Brazil.; ^4^Community Health Services, Grupo Hospitalar Conceição, Porto Alegre, Brazil.; ^5^School of Medicine, Universidade Federal do Rio Grande do Sul (UFRGS), Porto Alegre, Brazil.

**Keywords:** Primary Healthcare, Screening Programs, Quality, Quaternary Prevention, Brazil

## Abstract

**Background:** Cancer ranks second as a cause of death in Brazil. Although preventive practices are part of the daily routine of primary healthcare (PHC) teams, organized screening programs are lacking. This study aimed to evaluate the adequacy of preventive interventions in the main cancer types, as defined by the Brazilian government.

**Methods:** We analyzed cross-sectional data from a larger project conducted in 2016 with PHC service users and physicians from all over Brazil, interviewed by trained research staff. The sample was stratified by the number of PHC physicians per geographic region, who were eligible for inclusion if they had been working in the same PHC unit for at least one year. Twelve adult patients with at least two encounters were included per participating physician. Only the data from service users were analyzed in this study. We evaluated the questions about preventive practices and calculated the following indicators: coverage, focus, screening errors, and screening ratio. National guidelines and international evidence were used as a comparison parameter.

**Results:** The study population consisted of 6160 service users. The data indicate that the recommendations for cervical, breast, and prostate cancer screening and for treatment of tobacco dependence are not adequately followed. Coverage for breast and cervical cancer screening presented an overutilization bias, with rates 50% and 9% above the expected, respectively. The screening focus was also inadequate: 24%, 47%, and 54% of the screening tests for the three cancer types were performed in individuals outside the recommended age range. 31% of smokers were not approached for treatment.

**Conclusion:** These findings indicate that the Brazilian population has been subjected to inadequate and potentially iatrogenic interventions in PHC. New policies based on stricter criteria of adequacy and increased use of the concept of quaternary prevention may improve the effectiveness and equity of the health system.

## Background

**Key Messages**

** Implications for policy makers**

- Cancer screening actions of Family Health Strategy (FHS) teams, Brazilian primary care, have not followed evidence-based recommendations.
- The approach to smokers in primary care is below the expected standard, given the known effectiveness of treatment.
- Cancer screening tests and tobacco cessation programs are undertaken by FHS teams, Brazilian primary care, and must be monitored.
- Managers should implement continuing education actions to improve primary care delivery, focusing on patient safety and quaternary prevention.
- Financial incentives related to quality indicators of screening actions may help change the clinical practice of primary care teams.

** Implications for the public**

Preventive actions are part of health services offered by primary healthcare (PHC) professionals. This study investigated how cancer prevention actions have been undertaken by PHC teams in Brazil. The results show that Brazilian PHC teams have not followed official recommendations, and many service users have been subjected to actions that can cause more harm than good, in addition to reducing the efficacy of the health system. On the other hand, preventive actions such as the treatment of tobacco dependence, which should be widely recommended due to its clear benefits, are only rarely offered. Therefore, it is imperative that the country’s managers take actions to mitigate this problem. PHC professionals need to receive support for clinical decision-making and access to continuing education on topics such as screening, quaternary prevention, and patient safety.

Cancer ranks second as a cause of death in Brazil, and an estimated 625 000 new cases will be diagnosed in the year 2020.^1^ In this area, Brazil faces a challenge similar to that of the triple burden of disease, when the country needs to organize the health system to assist people with infectious diseases, chronic diseases, and external causes (violence and accidents), which is a typical characteristic of developing countries. That is, while cancers typical of developed countries are among the most prevalent ones in Brazil, the country still has high rates of cancer of the cervix, stomach, and esophagus, which are more common in low-income and middle-income countries. Except for non-melanoma skin cancer, the five most prevalent cancers in women are of the breast, colon/rectum, cervix, lung, and thyroid. Among men, the most prevalent is prostate cancer, followed by colorectal, lung, stomach, and oral cavity cancers.^1^

 Organized screening programs can have a positive impact on cancer-specific mortality and improve quality of life in some types of cancer.^2^ However, the effectiveness of an organized screening program is dependent on the quality of each process step: selection of the target population, choice of screening method and interval defined by the best evidence, establishment and audit of quality standards, continuous monitoring and evaluation, and adequate infrastructure and financial resources. Without these conditions, screening poses a potential risk of overdiagnosis and overtreatment for patients, and the program becomes inefficient for the health system.^3^ Organized screening programs with a positive effect include screening for cervical cancer, colorectal cancer, and less robustly, screening for breast cancer.^3-5^

 Two opportunistic cancer screening programs are currently available in Brazil: for cervical cancer and breast cancer.^6-8^ These programs are considered opportunistic because they partially meet the requirements systematized by the International Agency for Research on Cancer of the World Health Organization (WHO), as they mainly fail in actions related to monitoring and evaluation.^9-11^ In addition to screening programs, over the past 25 years the country has implemented other public policies in the fight against cancer, mostly in an isolated manner. These policies include smoking-related interventions, which were responsible for reducing the prevalence of smokers from 35% in 1989 to 11% in 2014, with an expected decrease in the incidence of lung cancer in the coming decades.^1,12^ In recent years, in addition to population actions, the country has intensified the treatment of tobacco dependence in primary healthcare (PHC) by expanding the purchase and distribution of medications, publishing clinical guidelines, and providing financial incentives for smoking cessation activities.^12^ Treatment of tobacco dependence is one of the most cost-effective health interventions and is directly related to the prevention of lung cancer, cardiovascular diseases, and pulmonary disorders.

 In Brazil, an important community-based approach to provide PHC is the Family Health Strategy (FHS). FHS teams are multidisciplinary healthcare teams organized geographically, covering areas of approximately 3500 patients each. The FHS was designed to reflect best practices and to facilitate first-contact care by locating PHC teams close to people’s homes, and the strategy currently covers 63% of the Brazilian population.^13,14^ A recent study found that the third most common reason for encounter in Brazilian PHC is related to seeking preventive medicine.^15^ Therefore, preventive interventions are part of the daily routine of the FHS teams, including cancer screening programs and treatment of tobacco dependence.^15,16^ A consequence of the large number of encounters with a preventive focus is the overuse of medical interventions. These interventions, in turn, also have consequences that range from individual harms,^17,18^ generated by false-positive results, overdiagnosis, and overtreatment,^19,20^ to the production of inequities in health systems (collective harms).

 Brazil currently faces an economic crisis, exacerbated by the pandemic, with an expected decrease in public financing of healthcare and a natural continuous increase in expenditures, which may further aggravate the chronic underfunding of the Brazilian public health system. It is therefore more than ever necessary that the government prioritize effective and efficient public policies based on the best available evidence.

 Given the great impact of cancer on the morbidity and mortality rates of the country, the high frequency of encounters in PHC for prevention, the evidence supporting interventions for early cancer detection and smoking cessation, and the country’s economic crisis scenario, the aim of the present study was to evaluate the adequacy of requests for screening tests in relation to the Brazilian government’s clinical guidelines and the provision of treatment of tobacco dependence by the FHS teams. We hypothesized that about 30% of requests for screening tests would be inadequate.

## Methods

 This study analyzed cross-sectional data from a larger project evaluating the *Mais Médicos* (More Doctors) Program. The data were collected nationwide from July to December 2016, and the main purpose of the survey was to assess whether the FHS teams are oriented (aligned) with the principles and attributes of PHC.^21^ The Primary Care Assessment Tool validated in Brazil (PCATool-Brazil) was used to survey PHC service users.^22,23^

 Briefly, PHC units across the country were divided into the five Brazilian geographic regions proportionally to the number of physicians with and without residency. Physicians and service users were approached by the researchers, who informed them of the study purpose and explained that participation was voluntary. Physicians were eligible for inclusion if they had been working in the same PHC unit for at least one year. Twelve consecutive adult patients (aged ≥18 years) with at least two encounters were included per participating physician and made up the sample of service users. A sample size of 6193 service users (516 physicians) was required to detect a difference of 0.3 points in PCATool-Brazil total score, with a power of 80% and 5% significance, given an anticipated dropout rate of 20%. Written informed consent was obtained from all participants prior to inclusion in the study.

 Data were collected by trained interviewers using an electronic device for data entry. Face-to-face interviews were conducted with both service users and physicians, but only the data from service users were analyzed in the present study. A structured questionnaire was administered to the service users, followed by the short version of the PCATool-Brazil for adult users. The structured questionnaire contained the questions that generated the data analyzed in the present study, which are described in Box 1, and also included information on socioeconomic and health status, utilization of PHC services, and type of care received in the PHC unit. These questions were developed based on questionnaires previously used in large national surveys (eg, National Household Sample Survey).^24^

**Box 1. Questions From the Service User Questionnaire Used in the Present Study**

- In the past 12 months, did [*name of the doctor*] perform a Pap smear or ask you if you had a Pap smear?
- In the past 12 months, did [*name of the doctor*] request a mammogram?
- In the past 12 months, did [*name of the doctor*] request a transvaginal ultrasound?
  - If so, when the transvaginal ultrasound was requested, did you have any health complaints such as pain or vaginal bleeding?
- In the past 12 months, did [*name of the doctor*] request a prostate blood test, the PSA test?
  - If so, when the PSA test was requested, did you have any urinary symptoms such as weak urinary stream, difficulty urinating, or a feeling of incomplete bladder emptying?
  - If so, do you use any medication for the prostate (eg, doxazosin/carduran/duomo or finasteride/calvin/flaxin)?
- In the past 12 months, did [*name of the doctor*] ask you if you smoke?
- Do you smoke?
  - If so, did [*name of the doctor*] recommend you stop smoking?

----------------

Abbreviation: PSA, Prostate-specific antigen.

In order to assess the adequacy of interventions related to cervical, breast, and prostate cancer screening, the following indicators were calculated: (*a*) Coverage (proportion of individuals screened among those with an indication – age defined for screening); (*b*) Focus (proportion of individuals with an indication according to age group among the total screened); (*c*) Screening errors: overscreening (tests in individuals with no indication among the total number of individuals outside the screening population) and underscreening (tests not requested for individuals meeting criteria among the total number of individuals meeting criteria); and (*d*) Screening ratio (percentage of screening tests requested for people meeting criteria to the percentage of screening tests requested for those not meeting criteria).^25,26^

 We performed a review of the official Brazilian guidelines and evidence from the literature on the screening of these four types of cancer and on the screening and treatment of tobacco dependence. Based on this review, we defined the population parameters that were used to assess the adequacy of the results. These parameters are summarized in Table 1.

**Table 1:** Parameters for Performing Cancer Prevention Activities in Brazilian PHC Based on National Clinical Guidelines and Best Available Evidence, Brazil, June 2019

	**Cervical Cancer**	**Breast Cancer**	**Ovarian Cancer**	**Prostate Cancer**	**Smoking**	**Treatment of Tobacco Dependence**
Indication for screening/treatment	Yes, organized	Yes, organized	No	Only when requested by the patient with informed/shared decision-making	Yes, opportunistic	Yes
Age range	Women aged 25-64 years (included)	Women aged 50-69 years (included)	Not applicable	Men aged 55-69 years (included)	Entire population	Entire smoking population
Frequency	Every 3 years	Every 2 years	Not applicable	Not applicable	At every opportunity	At every opportunity
Source	INCA,^8,27-29^ CTFPHC, USPSTF, Cochrane	INCA,^4,30-32^ CTFPHC, USPSTF, Cochrane	INCA,^33-35^ CTFPHC, USPSTF	INCA,^36-39^ CTFPHC, USPSTF, Cochrane	MoH,^40^ WHO,^41^ CTFPHC, Cochrane^42,43^	MoH,^40^ WHO,^41^ CTFPHC, Cochrane^42,44^

Abbreviations: PHC, primary healthcare; INCA, Brazilian National Cancer Institute; CTFPHC, Canadian Task Force on Preventive Healthcare; USPSTF, United States Preventive Services Task Force; Cochrane, Cochrane Database of Systematic Reviews; MoH, Brazilian Ministry of Health; WHO, World Health Organization.

 The authors decided to include in this article the evaluation of the request for transvaginal ultrasound as a screening tool for ovarian cancer despite the lack of evidence and guidelines to support screening for this cancer, based on the hypothesis that this was a common practice in PHC services.

 A descriptive analysis was performed using PAWS Statistics for Windows, version 18.0. Tables and charts were created in Excel Office 2010. Service users with incomplete data were excluded from the analysis.

## Results

 The present study population consisted of 6160 PHC service users who were interviewed in the umbrella project from July to December 2016, which accounted for 99.5% of the required sample size. Table 2 shows the characteristics of the study population in comparison with the Brazilian general population. Of the total number of women evaluated for cervical, breast, and ovarian cancer screening, 18, 13, and 6, respectively, were excluded from the analyses due to incomplete data. Of the total number of men evaluated for prostate cancer screening, 15 were excluded for the same reason. Regarding smoking, 54 service users had incomplete data.

**Table 2:** Characteristics of the Interviewed PHC Service Users (Study Population) in Comparison With the Brazilian General Population, Brazil, July 2016

**Variable**	**Study Population ** **(N = 6160)**	**General Population ** **(N = 204 860 000)**
Gender, No. (%)		
Female	4667 (75.8)	105 452 (51.1)
Male	1493 (24.2)	99 408 (48.9)
Race, n (%)		
Non-white	4105 (66.6)	112 220 (54.9)
White	2055 (33.4)	92 636 (45.1)
Socioeconomic status^a^, No. (%)		
A, B, C	3528 (57.3)	146 884 (71.7)
D, E	2632 (42.7)	57 976 (28.3)
Region of the country, No. (%)		
North	722 (11.7)	17 524 (8.5)
Northeast	2194 (35.6)	56 641 (27.6)
Southeast	1826 (29.6)	85 916 (42.0)
South	1043 (16.9)	29 290 (14.4)
Midwest	375 (6.1)	15 489 (7.5)
Age group (y), No. (%)		
<17	Not applicable	55102 981 (29.8)
18-24	677 (11.0)	24 132 759 (11.8)
25-44	2008 (32.6)	64 797 091 (31.6)
45-64	2291 (37.2)	43 341 200 (21.1)
65-79	1015 (16.5)	14 035 797 (6.9)
≥80 years	169 (2.7)	3 746 759 (1.8)

Abbreviation: PHC, primary healthcare. ^a^A, upper class (elite); B, upper middle class; C, lower middle class; D, working class; and E, poor and unemployed. Classes are defined according to the Brazilian Institute for Geography and Statistics.

 The results for cervical, breast, and prostate cancer screening are shown in Tables 3 and 4. The screening coverage rate of individuals in the correct age range was 66.0% for cervical cancer, 59.0% for breast cancer, and 62.0% for prostate cancer. The screening tests performed were appropriate for age in 76.8% of cases for cervical cancer, in 53.6% for breast cancer, and in only 46.8% for prostate cancer.

**Table 3:** Absolute Number of Patients Eligible for Screening for Cervical Cancer, Breast Cancer, and Prostate Cancer and Number of Patients With Reports of Screening According To Age Group for Examination, Brazil, July 2016

	**Cervical Cancer**	**Breast Cancer**	**Prostate Cancer**
Total women/men^a^, n	4649	4654	1209
Women/men within the appropriate age range^a^, n	3336	1585	369
Women/men outside the appropriate age range^a^, n	1313	3069	840
Total number of people undergoing examination, n	2885	1759	496
Total number of people undergoing examination within the appropriate age range, No. (%)	2217 (76.8)	943 (53.6)	232 (46.8)
Total number of people undergoing examination outside the appropriate age range, No. (%)	668 (23.2)	816 (46.4)	264 (53.2)

^a^Men who did not report urinary symptoms or use of medications for prostate problems.

**Table 4:** Indicators of Adequacy of Cancer Screening in Family Health Strategy Teams, Brazil, July 2016

**Screening Type**	**Indicator** ^a^	**Percentage of Patients (%)**	**95% CI**
Cervical cancer	Coverage	66	64.4-67.6
	Focus	76	74.4-77.5
	Overscreening	50	48.3-51.6
	Screening ratio	1.3	0.8 - 1.7
Breast cancer	Coverage	59	56.6-61.4
	Focus	53	50.6-55.3
	Overscreening	25	23.6-26.4
	Screening ratio	2.4	1.8 - 2.9
Prostate cancer	Coverage	62	57.1-66.9
	Focus	46	41.6-50.3
	Overscreening	26	23.5-28.5
	Screening ratio	2.4	1.3 – 3.5

Abbreviation: CI, confidence interval. ^a^The formula for calculating each of these indicators is described in the Methods section.

 When analyzing the population that is not within the screening profile in terms of age and periodicity (overscreening), the results showed that, on average, tests were requested for 33.5% of this population. From a population perspective, none of the cancer types were underscreened.

 The results for screening ratio were lower than expected. Regarding cervical cancer, the results showed that screened women were only 1.3 times more likely to meet than not to meet the age-group criterion.

 Regarding ovarian cancer, due to the lack of evidence for its screening, evaluation measures were not calculated. The results showed that, in 49.5% of all transvaginal ultrasound scans requested, women denied any gynecological complaints, such as pain and vaginal bleeding, which indicates that tests were requested for asymptomatic women.

 The prevalence of smoking in the study population was 11%. The results showed that 33% of the interviewees were not asked about smoking habits in the past 12 months by health professionals in the PHC unit, which indicates underscreening. When analyzing only the group of people who reported being smokers, 31% did not receive recommendations to stop smoking (undertreatment).

## Discussion

 The main result of this study was that the recommendations of the Brazilian Ministry of Health for cervical, breast, and prostate cancer screening, as well as for the treatment of tobacco dependence, are not being adequately followed by the FHS teams. This is also a common finding in the international literature evaluating screening programs in low-income and middle-income countries.^45^

 The rates for screening coverage of individuals in the correct age range for cervical cancer and breast cancer were above the expected rates of 33% and 50%, respectively, from a population perspective, which indicates overscreening in the correct age range—using as a reference for periodicity, tests performed one year before the study. In prostate cancer there is no comparison parameter, as there is no indication for population screening. The screening focus (correct age) was inadequate for the three types of cancer. If the screening focus were calculated based on the periodicity recommended by national guidelines, the rates would be even lower: 38%, 45%, and 37% for cervical, breast, and prostate cancer, respectively.

 There is overuse of tests in the target population for cervical and breast cancer screening, a misleading focus of tests on people not meeting the predetermined criteria, exposure of non-target populations to overscreening, and poor guidance on treatment of tobacco dependence.

 Regarding breast cancer screening, the results are compatible with some data from the literature, such as the results of Tomazelli et al,^46^ Freitas-Junior et al,^47^ and Corrêa et al,^48^ who reported that only 51%, 54% and 51%, respectively, of screening mammograms performed in Brazil were within the recommended age range, a finding comparable to that reported in the present study (53%). However, conflicting results emerged when comparing for screening focus (appropriate age and periodicity). The present study found that 45% of the screening tests were adequate in terms of age and periodicity, against 32% reported by Tomazelli et al^46^ and Corrêa et al.^48^ The fact that periodicity was only estimated in the present study may explain the difference.

 As for cervical cancer screening, our findings are slightly different from the literature. The literature reports a deficit of screening tests,^49^ whereas the present study found an overuse of screening tests similar to that reported by Vale et al.^50^ This difference can also be explained by the different modes of calculation, in relation to both periodicity and actual need for screening, and by our data collection strategy, whose target audience was patients attending PHC units. Regarding the percentage of tests performed in the correct age range, the WHO recommendation is 80%^3^ and the official rate in Brazil is 79%^51^– the result found in the present study was slightly lower than these rates (76%) but comparable to that reported in national^50,52^ and international surveys.^53,54^

 Although there is no guidance on prostate cancer screening, our results show that patients have been screened for this type of cancer—a finding supported by the national report on prostate cancer of the Brazilian National Cancer Institute^55^ and by data from the study of Santiago et al.^56^ In addition, when performed, screening does not focus on the age group of patients who would most benefit from it, since more than 50% of the tests were performed in men outside the recommended age range. The percentage of men screened for prostate cancer (52%), regardless of age and symptoms, was comparable to that reported by Amorim et al.^57^ It is important to note that there are numerous guidelines in Brazil (from the executive branch, from the legislative branch, and from medical societies), and many of them contradict each other in relation to cancer screening, which makes decisions more difficult for health professionals and patients.

 Another important finding is that, on average, 33% of people who are not part of the target population of the national screening guidelines were subjected to unnecessary or low-value tests. Narrowing the focus to the target population is part of the actions undertaken to reduce overutilization and its harms in screening programs, as well as a goal to obtain better results from the programs. In addition, almost 50% of transvaginal ultrasound scans were requested for asymptomatic women. These findings are supported by evidence from international studies addressing the overuse of medical interventions and their hazards, including false-positive results, overdiagnosis, and overtreatment.^58,59^ This also highlights the lack of quaternary prevention and patient safety practices in PHC in Brazil.

 Overuse of medical tests can trigger a cascade of diagnostic studies with individual and systemic implications.^17^ For the health system, it implies less effectiveness resulting from the use of financial and technical resources in activities that do not generate benefits. For the population, in addition to the inefficient use of resources, there is a waste of time for the patient, who is subjected to unnecessary tests and exposed to risks, tangible and intangible, related to overscreening and, consequently, overdiagnosis and overtreatment.^46^ This finding also has an impact on the (symptomatic) population that needs to undergo diagnostic rather than screening tests, as they end up competing with each other for resources that are scarce and poorly allocated. Inappropriate use of finite resources reduces the supply of tests available for those who really need them, leading to inefficiency of the health system.

 This study indicates that the population served by the FHS teams has been subjected to excessive and potentially iatrogenic interventions. In this respect, practices related to quaternary prevention should be encouraged in PHC to prevent harms and to increase the effectiveness and equity of the Brazilian health system.^17^

 Regarding smoking, the Brazilian National Health Interview Survey reports a prevalence of smokers of 14.7%,^60^ whereas the present study found a slightly lower rate of 11%; however, our study had a service-based rather than a population-based approach. Smoking has been associated with one-third of all cancer deaths, and both individual and population-based interventions on cessation are essential actions given their impact and cost-effectiveness in the control of cancer, cardiovascular diseases, and chronic lung diseases.^61^ In view of this evidence, it is noteworthy our finding that 30% of smokers were not approached by the FHS teams for treatment, which leaves room for further qualification of the teams and expansion of access to treatment of tobacco dependence, considering that the national policy determines that this is the responsibility of PHC.

 The results of the present study show that screening approaches are used by physicians based on their own discretion in the medical consultation rather than on the definition of the PHC service. This decision appears to be made at an individual (rather than population) level, disregarding whether individual characteristics are outside or within the criteria of the Brazilian Ministry of Health or best-evidence standards.

 The limitations of this study include its cross-sectional design and the impossibility of statistically analyzing the relationship of our findings with the individual characteristics of service users, health professionals, or the service itself because the results evaluated population-based patterns. Additionally, recall bias may have been present due to the 12-month time-frame used for the questions on the tests performed. Loss of service users may have also reduced the representativeness of findings. In cervical and breast cancer screening, limitations included the lack of information on cancer-related symptoms and the interval used in the study, which is different from that usually used in this type of study. The level of external validity is debatable, as the study is limited to people enrolled in the FHS; it is not a nationwide population-based study. It is also important to consider that self-report data served as the basis for analysis. The research data on requests for screening tests were obtained from a single source (the patient), and it is known that patients tend to overestimate self-care practices when self-reported. However, we did not have access to patients’ medical records to confirm the data. The strength of the study is the production of novel results for the country.

## Conclusion

 The results of this first national survey, which addresses the quality of screening actions in the population enrolled in the FHS, show that the first step of organized screening programs (focused on the target population) has not been adequately followed. This reduces the likelihood of a protective effect against possible harms from unnecessary interventions.^3^

 The inadequacy of requests for screening tests and the low rates of recommendation for treatment of tobacco dependence indicate low-quality care in the FHS and reveal the iatrogenic potential of preventive interventions. Considering that prevention is the third most common reason for encounter in Brazilian PHC, it is essential that screening actions are the target of further research for a better understanding of this practice and its effects.

 It is also an ethical imperative that the country reassesses all programs related to early cancer detection.^18^ Expanding program monitoring and assessment is a key step for efficient implementation of organized screening programs.^43^

 The economic impact of such low-quality care should also be investigated so that the managers of the Brazilian public health system are provided with sufficient evidence to prioritize more cost-effective actions. In the current Brazilian scenario, implementing actions for early diagnosis and timely treatment can be more cost-effective than applying opportunistic screening programs.

## Ethical issues

 The study was approved by the Research Ethics Committee of Hospital de Clínicas de Porto Alegre (approval number 1.219.926).

## Competing interests

 Authors declare that they have no competing interests.

## Authors’ contributions

 Conception and design: PSC, EH, MRG, LH, SM, MA, RR, and LW. Acquisition of data: PSC, AD, RAMS, and LH. Analysis and interpretation of data: PSC, EH, MRG, SM, LH. Drafting of the manuscript: PSC. Critical revision of the manuscript for important intellectual content: EH, MRG, and SM. Statistical analysis: EH, LH, and PSC. Obtaining funding: EH. Administrative, technical, or material support: PSC, LH, AD, and RAMS. Supervision: EH. All authors have read and approved the final version of the article.

## Funding

 The research was funded by the Pan American Health Organization.
